# Integrated Systems Analysis of Mixed Neuroglial Cultures Proteome Post Oxycodone Exposure

**DOI:** 10.3390/ijms22126421

**Published:** 2021-06-15

**Authors:** Rahul S. Guda, Katherine E. Odegaard, Chengxi Tan, Victoria L. Schaal, Sowmya V. Yelamanchili, Gurudutt Pendyala

**Affiliations:** 1 Department of Anesthesiology, University of Nebraska Medical Center, Omaha, NE 68198, USA; rahulguda@college.harvard.edu (R.S.G.); katherine.odegaard@unmc.edu (K.E.O.); Chengxi.Tan@ucsf.edu (C.T.); vicki.schaal@unmc.edu (V.L.S.); syelamanchili@unmc.edu (S.V.Y.); 2 Child Health Research Institute, Omaha, NE 68198, USA

**Keywords:** oxycodone, synaptogenesis, neuroglial culture, opioids, bioinformatics

## Abstract

Opioid abuse has become a major public health crisis that affects millions of individuals across the globe. This widespread abuse of prescription opioids and dramatic increase in the availability of illicit opioids have created what is known as the opioid epidemic. Pregnant women are a particularly vulnerable group since they are prescribed for opioids such as morphine, buprenorphine, and methadone, all of which have been shown to cross the placenta and potentially impact the developing fetus. Limited information exists regarding the effect of oxycodone (oxy) on synaptic alterations. To fill this knowledge gap, we employed an integrated system approach to identify proteomic signatures and pathways impacted on mixed neuroglial cultures treated with oxy for 24 h. Differentially expressed proteins were mapped onto global canonical pathways using ingenuity pathway analysis (IPA), identifying enriched pathways associated with ephrin signaling, semaphorin signaling, synaptic long-term depression, endocannabinoid signaling, and opioid signaling. Further analysis by ClueGO identified that the dominant category of differentially expressed protein functions was associated with GDP binding. Since opioid receptors are G-protein coupled receptors (GPCRs), these data indicate that oxy exposure perturbs key pathways associated with synaptic function.

## 1. Introduction

The opioid epidemic has solidified itself as a national emergency. While past surges in opioid-related deaths were primarily due to illicit opioids, such as heroin, synthetic opioids have ushered in a new era of opioid addiction, accounting for over 67% of opioid overdose deaths in 2018 [1]. Oxy, a semisynthetic prescription opioid, is among those creating this new wave of opioid-related deaths. Despite having approximately one-tenth the potency of morphine [2], oxy has demonstrated a comparable ability to cross the blood–brain barrier (BBB) and inflict neuronal damage [3]. Further, previous studies have identified that offspring born to opioid-exposed mothers exhibit a number of deficits in development and behavior, as well as molecular alterations, impacting synaptic signaling [4]. Oxy exposure, specifically, has been shown to impact the expression of miRNA cargoes within extracellular vesicles, creating a potential for impaired patterns of neurodevelopment [5]. Additionally, pre- and postnatal oxy exposure has been shown to have a generational impact on behavioral alterations and the expression of key genes associated with synaptic signaling [6]. Recently, our group has utilized electrophysiological studies to elucidate synaptic alterations associated with pre- and postnatal oxy exposure, identifying changes in excitatory postsynaptic signaling in the postnatal exposure group [7]. 

With the recent evidence of the effects of oxy exposure on synaptic function in vivo, we further sought to investigate these changes in vitro. The present study focused on identifying key synaptic signatures using quantitative mass spectrometry-based proteomics post oxy exposure on mixed neuroglial cell cultures, comprising predominantly of astrocytes and neurons. Next, using bioinformatics tools, we identified pathways associated with neurotransmitter signaling, neurodevelopment, and nervous system signaling to be dysregulated. In summary, our current study using a systems approach provides, for the first time, a comprehensive characterization of oxy exposure on key pathways associated with modulating brain development and function.

## 2. Results

### 2.1. Purity of Mixed Neuroglial Cultures

**Figure 1:**
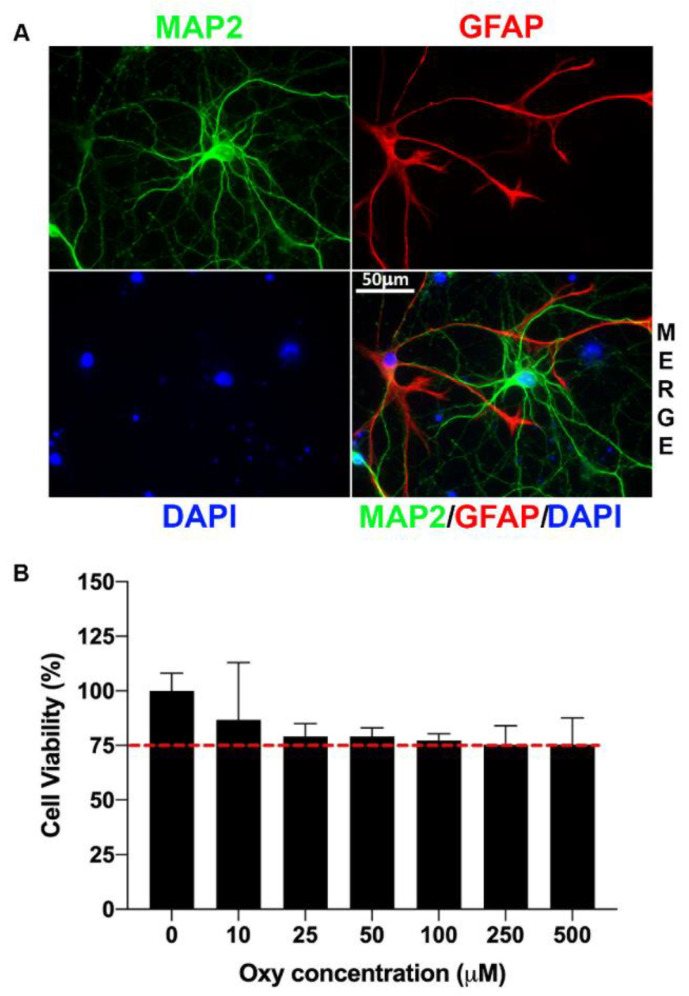
Mixed neuroglial culture purity and oxy dose selection. (**A**) Purity of mixed neuroglial cultures as evidenced by immunolabeling with MAP2 (neuronal marker) and GFAP (astrocyte marker). (**B**) Results of MTT assay for optimal selection of oxy concentration to be used in further in vitro studies. Oxy concentrations of 250 and 500 μM resulted in >75% cell death; 100 μM, which represented the highest concentration of oxy resulting in the least cell death, was used for our in vitro experiments. Data shown are from three independent experiments.

Astrocytes play a pivotal role in exchanging information with synaptic neuronal elements to regulate synaptic activity and neurotransmission, thus giving rise to the new concept of a “tripartite synapse” [8]. Mixed neuroglial cultures comprised predominantly of neurons and astrocytes have been used to investigate the tripartite synapse concept. In this current study, mixed neuroglial cultures were isolated from embryonic day 18 (E18) pups. After growing these cultures until DIV14 (days in vitro), we validated the purity of our mixed cultures through immunolabeling with the neuronal and astrocyte markers MAP2 and GFAP, respectively. As seen in Figure 1A, there was definitive staining for these two markers as determined by confocal imaging, thus denoting the purity of our mixed neuroglial cultures.

### 2.2. Cell Death Assay

To ascertain the optimal physiological concentration of oxy where cell death was not acutely prominent, we assessed cell viability over a broad range of oxy concentrations. Results from our MTT assay showed that oxy concentrations of 250 and 500 μM resulted in >75% cell death (Figure 1B). Accordingly, 100 μM, which represented the highest concentration of oxy resulting in the least cell death, was chosen for our studies. 

### 2.3. Characterization of the Altered Proteome Upon Oxy Exposure

**Figure 2:**
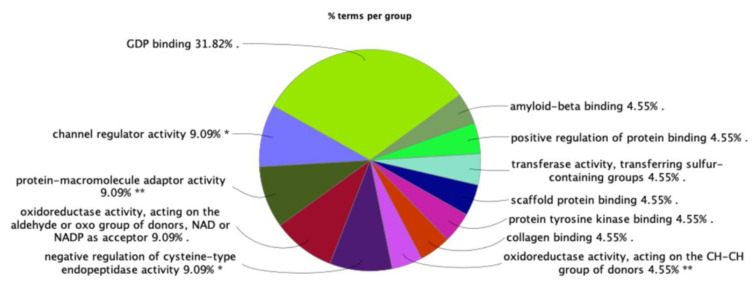
Distribution of functional categories of gene ontology (GO) terms associated with DEPs in the oxy-exposed neuroglial cultures. The size of each category within the pie chart represents the percentage of included terms. All enriched GO terms are statistically significant. Single (*) or double (**) asterisk indicate significant enriched GO terms at the *p* < 0.05 and *p* < 0.01 statistical levels, respectively.

Proteomics data from mass spectrometry studies were analyzed to identify differentially expressed proteins (DEPs) in the oxy versus control groups. Using a combined threshold of ±1.5 fold-change and *p* < 0.05, 173 upregulated and 9 downregulated proteins were selected and advanced for functional characterization (Supplementary Table S1). The molecular functions of these DEPs were identified by ClueGO analysis tool. A total of 182 DEPs were mapped to 173 unique genes in ClueGO; of these, 144 genes (83%) had functional annotations with experimental evidence. The most dominating functional group was the GDP (guanosine 5′-diphosphate) binding function, with 31.82% of the GO terms associated with this function, followed by channel regulator activity, protein-macromolecular adaptor, and oxidoreductase activity, among others (Figure 2). GDP binding proteins, small GTPases belonging to the Ras superfamily, and membrane proteins associated with the regulation of ion channel activity constituted the dominant group of DEPs in the oxy-exposed group compared to the control. A list of GO terms and associated genes can be found in Supplementary Table S2.

**Figure 3:**
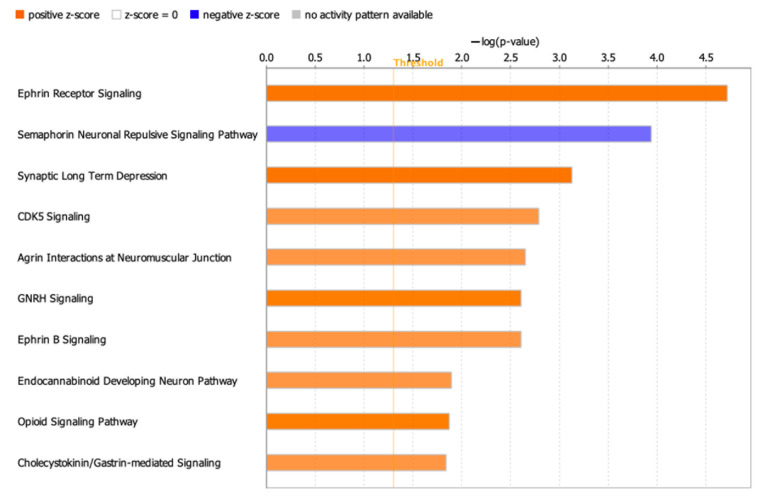
Enriched pathways associated with neurodevelopment and nervous system signaling in the oxy-exposed neuroglial cultures. Orange bars indicate upregulated pathways and blue denote downregulated. All pathways are significant with *p* < 0.05 and an absolute *z*-score ≥ 1; the threshold line corresponds to *p* < 0.05.

Pathway mapping of DEPs using IPA showed upregulated pathways associated with sirtuin signaling, respiration, and cholesterol biosynthesis; downregulated pathways were associated with semaphorin neuronal repulsive signaling and phosphatase and tensin homolog (PTEN) signaling (Supplementary Table S3). Because these results show only the globally affected pathways, we further investigated the specific effects of the oxy treatment on neurodevelopmental and signaling pathways using the Neuro-47 pathways from IPA (Supplementary Table S4). As shown in Figure 3, pathways associated with ephrin receptor, CDK5, GNRH, semaphorin neuronal repulsive signaling pathways, synaptic long-term depression, agrin interactions at neuromuscular junction, endocannabinoid developing neuron, and opioid signaling pathways were highly enriched. Interestingly, the semaphorin neuronal repulsive signaling was the only downregulated pathway in the oxy-exposed neuroglial cultures. Genes affecting the downregulation of this pathway included *Cd44*, *Erbb2* (Erb-B2 receptor tyrosine kinase 2), integrin subunit alpha 1 and 2 (*Itga1* and *Itgb1*), *Map2k1* (mitogen-activated protein kinase kinase 1), *Ppp1r12a* (protein phosphatase 1 regulatory subunit 12A), and *Rras* (related to RAS viral oncogene homolog) (Supplementary Table S3).

**Figure 4:**
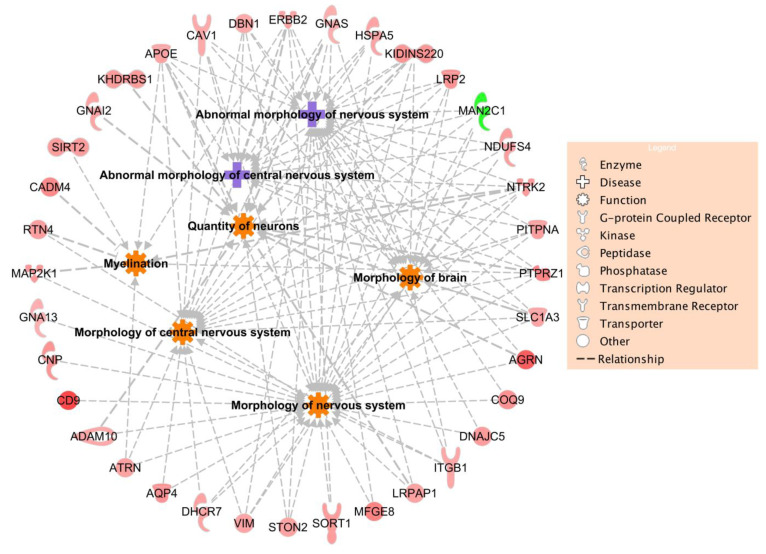
Differentially expressed proteins and their associations with multiple neurodevelopmental systems. Shapes of the molecules denote specific functions, such as G-protein coupled receptor, kinase, transcription regulator, and transporter.

In the process of determining the relationship between DEPs in the oxy-exposed group and nervous system development, IPA mapping of DEPs identified a set of 36 genes that were causative of various abnormal morphologies of the central and peripheral nervous system, quantity of neurons, myelination, and morphology of the brain. Several genes, including *Cav1*, *Gnas*, and *Ntrk2*, are implicated in multiple processes and have a broader influence on the overall neurodevelopmental system. The multiple roles played by these genes in various nervous system development processes is depicted in Figure 4. Gene-to-disease associations are provided in Supplementary Table S5.

**Figure 5:**
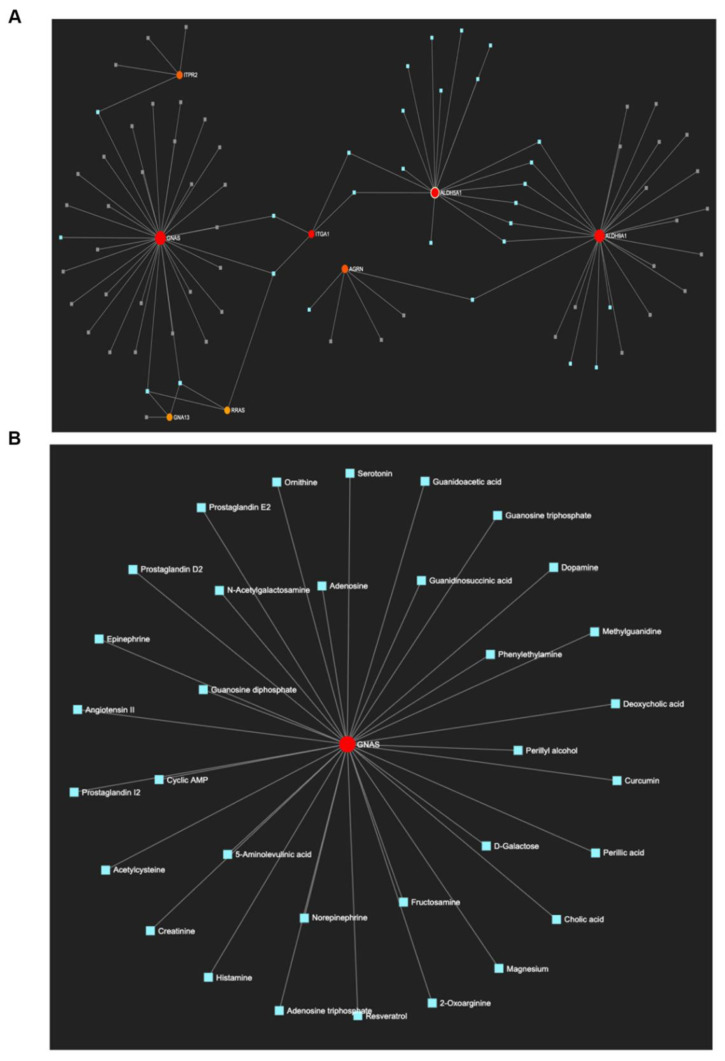
Gene–metabolite associations for selected DEPs involved in the enriched pathways of oxy-exposed neuroglial cultures. (**A**) Overall gene–metabolite network. (**B**) Highlighted network of metabolites associated with the GNAS gene locus.

We further investigated gene–metabolite associations using Metaboanalyst on a unique set of 17 genes associated with pathway enrichment in oxy-treated samples. As shown in the metabolite interaction network in Figure 5A, eight out of the seventeen genes had shared metabolites, indicating interactions and mutual regulatory effects in neurodevelopment and signaling pathways. GNAS complex locus (guanine nucleotide-binding protein or G protein) showed the highest metabolic connectivity, followed by aldehyde dehydrogenase family members (ALDH5A1 and ALDH9A1). A detailed list of all metabolites is provided in Supplementary Table S6. Further investigation of the metabolites associated with GNAS complex revealed several neurotransmitters, including dopamine, serotonin, and histamine (Figure 5B). 

## 3. Discussion

To date, there is a lack of in vitro studies examining molecular-level changes associated with oxy exposure in neuroglial cultures. The present bioinformatic analyses have shed light on this avenue of study, identifying several differentially regulated genes and the subsequently affected pathways. After verifying our purified neuroglial cultures through immunolabeling, we sought to determine which concentration of oxy would best suit our study. In the literature, existing studies have used a range of oxy concentrations for their in vitro culture work [9,10,11,12,13,14]. Of these concentrations, the most pertinent range is from 0 to 10 µM, which correspond with plasma concentrations in the low micromolar range [12]. As our study is the first to consider a neuroglial model in the context of oxy exposure, we extended this range of concentrations into higher micromolar concentrations and selected our oxy concentration (100 µM) based on cell death. 

From our proteomic and bioinformatic analyses, we found that GDP binding was the dominant molecular function category affected in our study. The G proteins involved in G-protein-coupled receptors (GPCRs) like opioid receptors [15] have an important role as molecular transducing elements that couple membrane receptors to their molecular effectors and regulate the gating of ion channels affecting the membrane potential of target cells [16]. Differentially expressed genes associated with GDP binding included GNAI2 and GNAI3 (G protein subunits alpha i2 and i3), and members of Ras superfamily of small GTPases (RRAS, RAB14, RAB5A, RAB6A). GNAI2 and GNAI3 are abundantly expressed in immune cells [17] and GNAI3 has been shown to regulate cytokine responses [18,19]. Interestingly, GNAI2 has been shown to negatively regulate GNAI3, impacting the activation of Akt, mTORC1, and ERK1/2 involved in epidermal growth-factor-related processes, such as cell growth, migration, and survival [20,21]. Intriguingly, mTORC1 is required for the initiation and maintenance of chronic pain and opioid-induced tolerance/hyperalgesia [22], so the further investigation of these G protein subunits and their involvement in mTORC1 signaling may provide insight into these effects of opioid exposure. The Ras superfamily of small GTPases also warrant further investigation, as it has been shown to have a critical role in several processes, including neurogenesis, differentiation, gene expression, membrane and protein trafficking, vesicular trafficking, and synaptic plasticity [23]. 

Several highly relevant pathways, such as sirtuin signaling, ephrin signaling, and semaphorin signaling, were enriched in our study. Our findings revealed alterations in sirtuin signaling that may stem from an upregulation of SIRT2. SIRT2 is associated with processes such as carcinogenesis, infection, cell survival, DNA damage, and cell cycle regulation [24]. SIRT2 has also been associated with central nervous system development, and its overexpression may reverse stress-induced depressive-like behavior, potentially through its regulation of neurogenesis [25]. Interestingly, upregulation of sirtuins (SIRT1/SIRT2) in the nucleus accumbens—the subcortical region of brain most implicated in drug addiction—has been observed in both morphine- and cocaine-administered mice [26]. Additionally, treatment with the opioids morphine and fentanyl has been shown to increase SIRT2 expression [27]. Ephrin receptor signaling, which has been implicated in the regulation of embryonic development, axon guidance [28], and cellular processes such as long-term potentiation in adulthood [29] was upregulated in the neuroglial cultures treated with oxy. In contrast, semaphorin signaling was downregulated in the oxy-exposed neuroglial cultures. Semaphorins provide repulsive or attractive cues for migrating cells and growing neurites, and this signaling regulates many development processes, including neuronal circuit assembly [30]. Semaphorins are particularly involved in maintaining the neuronal structure and function in the early postnatal and juvenile nervous system by regulating synaptogenesis, axon pruning, and the density and maturation of dendritic spines [31]. Intriguingly, a mouse self-administration model of oxy showed that chronic oxy use altered the expression of several ephrin and semaphoring genes associated with axon guidance [32]. We have also previously shown alterations in axon guidance systems in our study of pre- and postnatal oxy exposure [7]. The differential regulation of these pathways may contribute to impaired neuronal structure and synaptic transmission in oxy-exposed individuals.

Intriguingly, of the 36 DEPs identified in our IPA, only one gene, MAN2C1, was downregulated. MAN2C1-deficient mice have previously been shown to exhibit biochemical and histological alterations in white matter tracts [33]. Upregulated genes included CAV1, NTRK2, and GNAS, all of which are implicated in multiple processes and have a broader influence on the overall neurodevelopmental system. CAV1, an important scaffolding protein that binds to GPCRs and plays a role in regulating neurostructural plasticity, has been implicated as a molecular target for inhibiting morphine-induced neuroplasticity; morphine treatment increased Cav1 mRNA levels, and inhibition of Cav1 expression reduced morphine-induced increases in neuronal growth [34]. Chronic opioid treatment has also been shown to alter Cav1 scaffolding, suggesting its potential role in the development of opioid tolerance and dependence [35]. Structural changes in the developing human brain following acute and chronic morphine exposure may also be due to alterations in neuronal differentiation and neurite outgrowth genes like NTRK2 [36]. Indeed, NTRK2 levels have been shown to increase in the hippocampus of morphine-dependent rats [37]. With the involvement of GNAS in the activation of GPCRs like the opioid receptors, it has been suggested that GNAS may mediate the effects of morphine in the development of tolerance and dependence [38]. We further investigated the metabolite associations of GNAS, several of which were neurotransmitters and their precursors, suggesting the extensive involvement of the GNAS complex with addiction and nervous system signaling pathways. This gene-metabolite mapping analysis also revealed networks associated with aldehyde dehydrogenase genes, specifically ALDH9A1 and ALDH5A1. Interestingly, variants of ALDH5A1 have been shown to influence patient responses to methadone treatment, the predominant therapy for opioid dependence [39]. Although well-studied in alcohol dependence [40], more investigation into these aldehyde dehydrogenases is needed in the realm of opioid dependence.

In summary, our results have shown the enrichment of several relevant pathways, cellular processes, and gene-metabolite associations in mixed neuroglial cultures treated with oxy. Quantitative proteomics and thorough systems analyses revealed differentially expressed proteins from oxy vs. control groups associated with enriched protein functions and pathways relevant to neurodevelopment and nervous system signaling. Among the most important pathways were those involving GDP signaling, sirtuin signaling, ephrin signaling, and semaphorin signaling. Further analysis of pathway and gene-metabolite interactions for genes involved with neurodevelopment and signaling identified CAV1, NTRK2, GNAS, ALDH9A1, and ALDH5A1 as intriguing contenders for future study of oxy’s effects on synaptic structures and signaling, as well as opioid dependence in general. Overall, this in vitro study has provided insights into the potential effects of oxy treatment on synaptic development and signaling in vitro.

## 4. Materials and Methods 

### 4.1. Animals and Ethics Statement

Pregnant Sprague–Dawley rats were purchased from Charles River Laboratories Inc. (Wilmington, MA, USA) and housed in a 12 h light–dark cycle and fed ad libitum. All procedures and protocols (#17-080, 01/06/2021) were approved by the Institutional Animal Care and Use Committee of the University of Nebraska Medical Center and conducted in accordance with the National Institutes of Health Guide for the Care and Use of Laboratory Animals.

### 4.2. Isolation of Mixed Neuroglial Cells

Mixed cultures were established as described in Sanchez et al. [41]. Briefly, E18 embryonic brain cortices were resuspended in HBSS medium by gently pipetting up and down. The cell suspension was centrifuged for 5 min, 500× *g* at 4 °C, and the cell pellet was resuspended in DMEM/F12 with 10% FBS medium. The homogenous cell mixture was passed through a 70 µm cell strainer and cells were counted. Cells were then plated onto six-well plates at a density of 1 × 10^6^ cells per well. For immunolabeling, 2.5 × 10^5^ cells per well were plated on coverslips in a 24-well plate. For cell viability assays, 1 × 10^4^ cells per well were plated in a 96-well plate. After 48 h, the cell culture medium was replaced by complete Neurobasal media (Life Technologies Corporation, Carlsbad, CA, USA) containing B27 (Life Technologies Corporation) and pen–strep (Life Technologies Corporation) for 14 days. Half medium exchanges were performed every 2–3 days, and all experiments were performed after 14 days in vitro (DIV 14).

### 4.3. MTT Assay

Cell viability was measured by 3-(4,5-dmethylthiazol-2-yl)-2,5-diphenyl tetrazolium bromide (MTT) method, as described in our earlier published study [42]. DIV14 mixed cultures were seeded in 96-well plates and exposed to various concentrations of oxy (0, 10, 25, 50, 100, 250, and 500 μM) for 24 h. The concentration showing the least amount of cell death, as assessed by absorbance due to dissolution of the formazan crystals, was used for subsequent studies. For our study, we chose 100 μM oxy, which was the maximum dose that demonstrated a cell viability of >75%. 

### 4.4. Immunolabeling

Immunolabeling on formalin-fixed DIV14 mixed neuroglia cells was performed as described previously [5]. Briefly, cells were washed three times with PBS and then permeabilized with 0.25% Tween-20 for 15 min. Cells were then incubated in blocking solution consisting of 10% normal goat serum, 10% Tween-20, 10% BSA, and PBS for one hour, followed by antiMAP2 (1:500 rabbit, Invitrogen, Waltham, MA, USA) and antiGFAP (1:500 mouse, Sigma, St. Louis, MO, USA) at 4 °C overnight. The next day, cells were washed with PBS, followed by incubation with fluorophore-labeled secondary antibodies (1:500 chicken antirabbit Alexa488 for MAP2; 1:500 donkey antimouse Alexa568 for GFAP, Invitrogen, Waltham, MA, USA) and 100 µM DAPI (Life Technologies Corporation) for one hour. Cells were washed with 1× PBS and mounted with ProLong Gold antifade (Invitrogen P36930) on Superfrost Plus charged microscope slides (Fisherbrand, Ottawa, ON, Canada), followed by confocal imaging with a Zeiss Observer Z1 fluorescent microscope with ApoTome (63× oil objective, NA 1.4) available at the Advanced Microscopy Core Facility at UNMC.

### 4.5. Quantitative Proteomics

DIV14 control and 100 μM oxy-treated mixed neuroglial cultures were harvested in cold PBS. The cell pellet was lysed in RIPA buffer containing 1× protease and phosphatase cocktail inhibitor, followed by incubation on ice for 15 min. The samples were then sonicated three times at five-second intervals, followed by centrifugation at 10,000 RPM for 15 min at 4 °C. Protein quantification was carried out using Pierce BCA protein assay (Thermo Scientific, Rockford, IL, USA) as described in our earlier studies [6,7,42,43,44,45,46]. A total of 50 µg of protein per sample per group was taken, and detergent was removed by chloroform/methanol extraction. The protein pellet was resuspended in 100 mM ammonium bicarbonate and digested with mass spectrometry (MS)-grade trypsin (Pierce) overnight at 37 °C. Peptides were cleaned with PepClean C18 spin columns (Thermo) and resuspended in 2% acetonitrile (ACN) and 0.1% formic acid (FA). A total of 500 ng of each sample was loaded onto trap column Acclaim PepMap 100 75 µm × 2 cm C18 LC Columns (Thermo Scientific™) at a flow rate of 4 µL/min, then separated with a Thermo RSLC Ultimate 3000 (Thermo Scientific™) on a Thermo Easy-Spray PepMap RSLC C18 75 µm × 50 cm C-18 2 µm column (Thermo Scientific™) with a step gradient of 4–25% solvent B (0.1% FA in 80% ACN) from 10–130 min, and 25–45% solvent B for 130–145 min at 300 nL/min, and 50 °C with a 180 min total run time. Eluted peptides were analyzed by a Thermo Orbitrap Fusion Lumos Tribrid (Thermo Scientific™) mass spectrometer in a data-dependent acquisition mode. A full survey scan MS (from *m*/*z* 350–1800) was acquired in the Orbitrap with a resolution of 120,000. The AGC target for MS1 was set as 4 × 10^5^, and ion filling time set as 100 ms. The most intense ions with charge state 2–6 were isolated in 3 s cycle and fragmented using HCD fragmentation with 40% normalized collision energy and detected at a mass resolution of 30,000 at 200 *m*/*z*. The AGC target for MS/MS was set as 5 × 10^4^, and ion filling time was set as 60 ms, with dynamic exclusion set for 30 s with a 10 ppm mass window. Protein identification was performed by searching MS/MS data against the Swiss-Prot *Rattus norvegicus* protein database, using the in-house mascot 2.6.2 (Matrix Science, Boston, MA, USA) search engine. The search was set up for full tryptic peptides with a maximum of two missed cleavage sites. Acetylation of protein *N* terminus and oxidized methionine were included as variable modifications, and carbamidomethylation of cysteine was set as a fixed modification. The precursor mass tolerance threshold was set at 10 ppm, and maximum fragment mass error was 0.02 Da. The significance threshold of the ion score was calculated based on a false discovery rate of ≤1%. Qualitative analysis was performed using Progenesis QI proteomics 4.1 (Nonlinear Dynamics).

### 4.6. Bioinformatic Analyses

Differentially expressed proteins (DEPs) between oxy-treated and control DIV14 mixed neuroglial cultures were identified using both a fold change of ±1.5 and a *p*-value less than 0.05. Molecular functions of DEPs were identified with the ClueGO [47] plug-in Cytoscape (www.cytoscape.org, v3.8.2, accessed on 24 October 2020), using Gene Ontology category ‘Molecular Function’ with the evidence code ‘All_Experimental’. DEPs were mapped to global canonical pathways using Ingenuity Pathway Analysis (IPA, www.ingenuity.com, v01-16, accessed on 19 October 2020) software, using the ‘Core Analysis’ function. IPA takes as input the accession number of proteins, the fold change, or the *p*-value of protein expression between control and experimental groups, and computes the enriched pathways and interaction networks, among others, using IPA knowledgebase. DEPs that are causative of nervous system development diseases were mapped using ‘Diseases and Functions’ information in the IPA knowledgebase. Functional characterization of DEPs was carried out on a subset of 47 IPA pathways associated with ‘Neurotransmitters and Other Nervous System Signaling’ (referred in this study as Neuro-47). Gene and metabolite associations were mapped for a set of genes involved in the enriched pathways using the ‘network explorer’ module of Metaboanalyst software [48,49]. Metaboanalyst carries out integrative analysis of metabolomics data, where the network explorer module outputs metabolomic networks among a set of genes based on the literature knowledgebase.

### 4.7. Statistics

Data represented are from three to six independent experiments using the tests described in the text and figure legends. DEPs that had at least two unique peptides and a *t*-test *p*-value < 0.05 were considered significant. Tests were performed using Prism software (GraphPad Software Inc., San Diego, CA) for Macintosh. All statistical tests were performed and analyzed with GraphPad Prism (La Jolla, CA, USA); data are represented as mean ± SEM on the graphs.

## Data Availability

Data is contained within the article or supplementary material.

## Supplementary Materials

Supplementary Materials can be found at https://www.mdpi.com/article/10.3390/ijms22126421/s1. Table S1: Differentially expressed significant proteins; Table S2: ClueGO molecular functions; Table S3: IPA canonical pathways; Table S4: IPA canonical changed pathways; Table S5: IPA pathways; Table S6: Betweenness of key proteins.

## Abbreviations

Oxy	Oxycodone
BBB	Blood brain barrier
E18	Embryonic day 18
MAP2	Microtube associated protein 2
GFAP	Glial fibrillary acidic protein
μM	Micro molar
DEP	Differentially expressed proteins
GO	Gene ontology
IPA	Ingenuity pathway analysis
GNAS	GNAS complex locus
DIV	Days in vitro
MTT	(3-(4,5-Dimethylthiazol-2-yl)-2,5-Diphenyltetrazolium Bromide)
